# Editorial: Inflammatory muscle diseases: an update

**DOI:** 10.3389/fneur.2023.1259275

**Published:** 2023-08-08

**Authors:** Jantima Tanboon, Merrilee Needham, Tahseen Mozaffar, Werner Stenzel, Ichizo Nishino

**Affiliations:** ^1^Department of Pathology, Faculty of Medicine, Siriraj Hospital, Mahidol University, Bangkok, Thailand; ^2^University of Notre Dame Australia, Fremantle, WA, Australia; ^3^Institute for Immunology and Infectious Diseases, Murdoch University, Murdoch, WA, Australia; ^4^Department of Neurology, Fiona Stanley Hospital, Murdoch, WA, Australia; ^5^Department of Neurology, School of Medicine, University of California, Irvine, Irvine, CA, United States; ^6^Pathology and Laboratory Medicine, School of Medicine, University of California, Irvine, Irvine, CA, United States; ^7^School of Medicine, The Institute for Immunology, University of California, Irvine, Irvine, CA, United States; ^8^Department of Neuropathology, Berlin Institute of Health (BIH), Charité-Universitätsmedizin Berlin, Freie Universität Berlin, Humboldt-Universität zu Berlin, Berlin, Germany; ^9^Department of Neuromuscular Research, National Institute of Neuroscience, National Center of Neurology and Psychiatry (NCNP), Tokyo, Japan; ^10^Department of Genome Medicine Development, Medical Genome Center, National Center of Neurology and Psychiatry (NCNP), Tokyo, Japan; ^11^Department of Clinical Genome Analysis, Medical Genome Center, National Center of Neurology and Psychiatry (NCNP), Tokyo, Japan

**Keywords:** myositis, dermatomyositis, inclusion body myositis, immune mediated necrotizing myopathy, antisynthetase syndrome, imaging, immune checkpoint inhibitor, COVID-19

The evolution of classification of the idiopathic inflammatory myopathies (IIM) is fueled by myositis-specific antibodies (MSA), clinicopathological features, and discoveries in the “-omics” fields such as proteomics and transcriptomics. The major IIM in the current classification include dermatomyositis (DM), immune-mediated necrotizing myopathy (IMNM), antisynthetase syndrome (ASS), and inclusion body myositis (IBM) (1, 2). SARS-CoV-2 (COVID-19) infection-associated myopathy and immune checkpoint inhibitors-related myositis (iR-myositis) attract attention as emerging entities. The existence of polymyositis (PM) among the IIM has become controversial because most “cases” can be re-classified into other IIM, predominantly IMNM and ASS, using serological-clinical-pathological variables (3). Interestingly, PM with mitochondrial pathology (PM-Mito), which contains features resembling IBM except for rimmed vacuoles (i.e., endomysial inflammation, mitochondrial pathology, highly differentiated cytotoxic T cells, and type 2 interferon (IFN2, IFN-γ) pathway upregulation) has been re-recognized and proposed as early IBM (eIBM) in IBM-spectrum disease (4, 5). Many of the IIM are paradigmatically associated with certain MSA, i.e., DM with DM specific antibodies (DMSAs) including anti-TIF1-γ, anti-Mi-2, anti-MDA5, anti-NXP-2, and anti-SAE antibodies; IMNM with anti-SRP and anti-HMGCR antibodies; ASS with anti-tRNA synthetase antibodies including anti-Jo-1, anti-OJ, anti-PL-7, anti-PL-12, anti-EJ, anti-KS, anti-Zo, anti-Ha, and anti-Ly antibodies; and IBM with anti-cN1A antibody. The clinicopathological features associated with the same MSA in adult patients could be different from juvenile patients (age < 18 years old); this is particularly true for jDM with TIF1-γ where cancer is never associated with the disease in contrast to adults where cancer association accounts for approximately 50% of patients. The serological-clinical-pathological features in association with human leukocyte antigen (HLA) haplotypes in different IIM subtypes have been summarized elsewhere (1, 2, 6–13).

Muscle imaging can assist and complement IIM diagnosis and long-term management. The most common modalities, including magnetic resonance imaging (MRI), ultrasonography, and positron emission tomography (PET) can identify typical patterns of individual skeletal muscle involvement and disease activity (14–16). Patterns of fat replacement and tissue edema recognized by T1-weighted and T2-weighted fat suppressed/short tau inversion recovery (STIR) MRI sequences are helpful for diagnosis. The Dixon MRI sequence measures muscle fat fraction, which quantitatively reflects disease activity and progression. Ultrasonography is more affordable and more practical for bedside evaluation, but its performance largely depends on the user's skills. Furthermore, it can only evaluate the superficial muscle layers. PET using different tracers specific for corresponding cellular mechanisms can demonstrate disease activity for each IIM. Fluorine-18 (^18^F)-labeled fluorodeoxyglucose PET (^18^FDG-PET) can identify patterns of muscle involvement, and detect concurrent interstitial lung disease and malignancy in IIM via glucose metabolism (17). ^11^C-labeled Pittsburgh compound B-PET (PIB-PET) (18, 19) and ^18^F-Flobetapir PET identify amyloid-beta in IBM (20). The use of PET imaging that utilizes a CD8 T lymphocyte-based ligand shows tremendous potential in preliminary studies in IBM (21). Electrical impedance myography (EIM), although mainly present in research settings, is a promising portable device that can operate in bedside settings. EIM quantitatively assesses disturbances in the electrical properties of diseased muscle using electrical current (22). The article by Zubair et al. summarizes the current state and future direction of these modalities in IIM.

The most prevalent IIM in juvenile patients is juvenile DM (jDM, 75% of juvenile IIM) followed by IMNM (2.9–21% of juvenile IIM) and rarely ASS; IBM is not present in this age group. Without serological information, juvenile IMNM risks being misdiagnosed as jDM due to its association with skin lesions or as inherited myopathy due to insidious muscle weakness and refractoriness to steroid treatment. Misdiagnosis and delayed proper treatment in IMNM likely lead to unreversible muscle damage. A recent article by Wang and Liang raises awareness of and discusses current information on juvenile IMNM.

Characteristic molecular pathway activations and their surrogate immunohistochemical markers have been identified in all major subtypes of IIM except for IMNM. The IFN1 signaling pathway is highly upregulated in DM and associated with sarcoplasmic myxovirus resistance protein A (MxA) expression; the finding is currently recognized as one of the diagnostic criteria for DM (23). The IFN2 signaling pathway is highly upregulated in IBM and to a lesser degree in PM-Mito and ASS; the upregulation can be demonstrated by surrogate markers e.g., HLA-DR (major histocompatibility complex, MHC class II) and guanylate-binding protein (GBP6) (5, 11, 24). Notably, GBP6 IHC positivity in a muscle biopsy containing highly differentiated cytotoxic T cells (highlighted by KLRG1, CD57, and PD1 expression) differentiates IBM from PM-Mito (5). The myopathological features of IMNM are less specific than the other IIM and pose diagnostic challenges in the absence of serology results. Although the upregulation of genes associated with the autophagy-lysosome pathway and diffuse p62 sarcoplasmic staining suggest autophagy pathway alteration in IMNM, the pattern can also present in non-immune mediated rhabdomyolysis (25, 26). In IMNM, muscle RING-finger protein-1 (*MuRF1*, a muscle-specific E3 ubiquitin ligase-associated muscle atrophy gene) is upregulated in atrophic myotubes induced by *in vitro* anti-SRP and anti-HMGCR antibodies treatment (27). Although *MuRF1* is upregulated in multiple muscle atrophy models and *MuRF1* knockout mice were not affected by glucocorticoids-induced atrophy (28, 29), Yang et al. recently demonstrated sarcoplasmic MuRF1 expression in regenerating myofibers of IMNM and DM but not in atrophic fiber. Whether MuRF1 upregulation in IMNM and DM is associated with mechanisms different from *in vitro* muscle atrophy models and whether the expression can be useful in IMNM diagnosis needs further exploration.

COVID-19-associated myopathy is likely caused by an immune-mediated mechanism rather than direct SAR-CoV-2 infection. This speculation is supported by autopsy studies in critically ill COVID-19 patients with myositis symptoms showing prominent myopathology without viral protein expression (30, 31) and an absence of angiotensin-converting enzyme 2 (ACE2, the protein essential for viral entry) expression on skeletal muscle (32). DM, IMNM, and ASS developing during or after SAR-CoV-2 infection has been observed. A recent case report by Niedzielska et al.. expands the spectrum of concurrent anti-Mi-2 DM in patients with SARS-CoV-2 infection whose condition improved after corticosteroid therapy. Whether SARS-CoV-2 infection directly induced myositis or triggered pre-existing asymptomatic conditions in these single case reports are debatable.

iR-myositis (in the context of immune checkpoint inhibition; ICI) is often associated with oculobulbar weakness, myocarditis, anti-acetylcholine receptor (anti-AChR) antibody, anti-striational antibodies, and MSA (33–35). This entity is frequently mistaken for myasthenia gravis. A transcriptomic study has classified ICIs-myositis into ICI-MYO1 associated with myocarditis and prominent inflammation in muscle biopsy; ICI-MYO2 with mild inflammation in muscle biopsy; and ICI-DM with DM myopathology, anti-TIF1-γ antibody, and IFN1 pathway upregulation (35). IFN2 and interleukin 6 (IL6) pathways are highly upregulated in ICI-MYO1 and ICI-DM. Since targeting the IL6 pathway is reported to improve other ICI-triggered immune adverse events, it could be useful for ICIs-myositis treatment.

## Conclusion

IIM consists of evolving heterogeneous entities which require multimodality approaches for diagnosis and appropriate management.

## Author contributions

JT: Conceptualization, Writing—original draft. MN: Writing—review and editing. TM: Writing—review and editing. WS: Writing—review and editing. IN: Writing—review and editing.
